# A lack of functional NK1 receptors explains most, but not all, abnormal behaviours of NK1R-/- mice^1^

**DOI:** 10.1111/gbb.12195

**Published:** 2015-02-01

**Authors:** A J Porter, K Pillidge, Y C Tsai, J A Dudley, S P Hunt, S N Peirson, L A Brown, S C Stanford

**Affiliations:** † Department of Neuroscience, Physiology and Pharmacology; ‡ Department of Cell and Developmental Biology, University College LondonLondon; § Department of Clinical Neurosciences (Nuffield Laboratory of Ophthalmology), University of Oxford, John Radcliffe HospitalOxford, UK

**Keywords:** 5-Choice Serial Reaction-Time Task, ADHD, attention, diurnal motor rhythm, epigenetics, hyperactivity, impulsivity, NK1 receptor, perseveration, *TACR1* gene

## Abstract

Mice lacking functional neurokinin-1 receptors (NK1R-/-) display abnormal behaviours seen in Attention Deficit Hyperactivity Disorder (hyperactivity, impulsivity and inattentiveness). These abnormalities were evident when comparing the behaviour of separate (inbred: ‘*Hom*’) wildtype and NK1R-/- mouse strains. Here, we investigated whether the inbreeding protocol could influence their phenotype by comparing the behaviour of these mice with that of wildtype (NK1R+/+) and NK1R-/- progeny of heterozygous parents (‘*Het*’, derived from the same inbred strains). First, we recorded the spontaneous motor activity of the two colonies/genotypes, over 7 days. This continuous monitoring also enabled us to investigate whether the diurnal rhythm in motor activity differs in the two colonies/genotypes. NK1R-/- mice from both colonies were hyperactive compared with their wildtypes and their diurnal rhythm was also disrupted. Next, we evaluated the performance of the four groups of mice in the 5-Choice Serial Reaction-Time Task (5-CSRTT). During training, NK1R-/- mice from both colonies expressed more impulsive and perseverative behaviour than their wildtypes. During testing, only NK1R-/- mice from the *Hom* colony were more impulsive than their wildtypes, but NK1R-/- mice from both colonies were more perseverative. There were no colony differences in inattentiveness. Moreover, a genotype difference in this measure depended on time of day. We conclude that the hyperactivity, perseveration and, possibly, inattentiveness of NK1R-/- mice is a direct consequence of a lack of functional NK1R. However, the greater impulsivity of NK1R-/- mice depended on an interaction between a functional deficit of NK1R and other (possibly environmental and/or epigenetic) factors.

Mice with functional ablation of the substance P-preferring (NK1) receptor gene (NK1R-/-) (de Felipe *et al.* 1998) express locomotor *hyperactivity*, compared with their wildtypes (Fisher *et al.* 2007; Herpfer *et al.* 2005; Yan *et al.* 2010). Typically, they also express a greater incidence of *premature responses* (a form of impulsivity), *%omissions* (failure to respond in the task, which can indicate inattentiveness) and *perseveration* (repetitive nose-pokes), when tested for the first time in the 5-Choice Serial Reaction-Time Task (5-CSRTT; Dudley *et al.* 2013; Yan *et al.* 2011).

Hyperactivity, impulsivity and inattentiveness are diagnostic criteria for Attention Deficit Hyperactivity Disorder (ADHD). The possibility that the abnormal behaviours of NK1R-/- mice echo those seen in ADHD patients is supported by an association between polymorphisms in, or near, the *TACR1* receptor gene (the human equivalent of the *Nk1r* gene) and increased vulnerability to ADHD (Sharp *et al.* 2014; Yan *et al.* 2010).

All these studies were carried out on homozygous wildtype and NK1R-/- mice, maintained as two inbred strains, which have been housed separately. Whereas this approach reduces overbreeding, there is a risk that genetic drift, or differences in the environment of the two inbred strains, such as maternal physiology or interactions with littermates, influence their behaviour as adults (Crews *et al.* 2004; Crusio *et al.* 2009; Sasaki *et al.* 2014; Tarantino *et al.* 2011). Here, we aimed to establish whether any of the differences in the behaviour of inbred NK1R-/- mice and their wildtype counterparts could be attributed directly to a lack of functional NK1R, alone, or whether additional (e.g. environmental and/or epigenetic) factors influence their phenotype. To investigate this possibility, we compared the behaviour of the homozygous progeny (wildtype and NK1R-/-) of inbred, homozygous breeding-pairs (‘*Hom*’ colony) with the behaviour of the homozygous progeny of heterozygous breeding pairs (‘*Het*’ colony), derived from the same wildtype and NK1R-/- mouse inbred strains.

First, we monitored the spontaneous motor activity of wildtype and NK1R-/- mice from the two colonies. Given the extensive evidence that the normal sleep / arousal pattern is disturbed in patients with ADHD (Kooij & Bijlenga 2013; van Veen *et al.* 2010), we were also interested in establishing whether the diurnal rhythm of motor activity is similarly disrupted in NK1R-/- mice. To that end, we used activity sensors that enabled us to monitor the movement of the mice over the entire 24 h cycle.

In a second series of experiments, we compared the performance of the two colonies and two genotypes in the 5-CSRTT: this procedure is widely used to evaluate animals' visual attention and response control (Robbins 2002). We aimed to determine whether the impulsivity, inattentiveness and perseveration of NK1R-/- mice reported previously (Dudley *et al.* 2013; Yan *et al.* 2010) is also evident in NK1R-/- mice bred from heterozygous parents.

## Materials and methods

These experiments were authorized under the UK Animals (Scientific Procedures) Act 1986 and received approval from the local Animal Welfare and Ethical Review Body at University College London.

### Animals

We studied two colonies of mice, both held at a facility at UCL and derived from a single colony of mice (129/Sv *x* C57BL/6J background strain, crossed with an outbred MF1 strain), that was developed many generations ago (described fully in: de Felipe *et al.* 1998). Given the constraint of the maximum number of mice that we could train and test each day in the 5-CSRTT (24), we opted to study males, only. This was partly because ADHD is more prevalent in males. However, we also needed to be able to compare the results of this experiment with those from all our previous studies in which we have compared the behaviour of the two male genotypes in the 5-CSRTT.

The first (‘*Hom*’) colony comprised two inbred homozygous lines: mice with functional ablation of the *Nk1r* gene (NK1R-/-; ‘*KO-Hom*’) and their wildtype counterparts (NK1R+/+; ‘*WT-Hom*’), which have been maintained as separate strains since their production (approximately 17 years ago). The second (‘*Het*’) colony derived from the same inbred (homozygous) strains after cross-breeding wildtype and NK1R-/- mice from the *Hom* strains to produce heterozygous offspring (F1). Heterozygous breeding-pairs were then used to produce litters containing wildtype (NK1R+/+; ‘*WT-Het*’), NK1R-/- (‘*KO-Het*’) and NK1R+/− mice (F2). Only the homozygous progeny were used in these experiments. The genotypes of the *WT-Het* and *KO-Het* (NK1R+/+ and NK1R-/-) mice were confirmed using tissue (ear-punch) samples and performing the polymerase chain reaction (PCR) followed by electrophoresis.

The two genotypes from the *Hom* colony were housed separately such that every home-cage contained up to four *WT-Hom* or four *KO-Hom* littermates. By contrast, the two (homozygous) genotypes from the *Het* colony were housed together as mixed litters, containing up to four *WT-Het* (NK1R+/+) / *KO-Het* (NK1R-/-) littermates (NK1R+/− mice were removed and culled at weaning (age 3 weeks)). In the *Het* colony, cages contained at least one mouse of each genotype. Other aspects of housing and husbandry were the same for the two colonies. Briefly, both colonies were housed in the same holding room at 21 ± 2°C, 45 ± 5% humidity, with a 12:12 h light:dark cycle (lighting increased in steps from 0700 h to 0800 h and reduced in steps from 1900 h to 2000 h). The home-cages incorporated the same environmental enrichment and sawdust bedding (3Rs Bedding Pty, Ltd., London W2, UK) and were cleaned twice weekly. Water and food [2018 global Rodent Diet (Harlan)] were freely available for mice used to monitor activity in the home cage, but mice destined for the 5-CSRTT were subject to restricted diet (see below).

### Activity monitoring

Five male mice, aged 8–14 weeks at the start of testing, were used from each group (*WT-Hom*, *KO-Hom*, *WT-Het*, *KO-Het*). Mice from the *Hom* colony were taken from two separate breeding-pairs for each genotype, while mice from the *Het* colony were taken from three separate breeding-pairs. Animals were age-matched across the four experimental groups as closely as possible. Between 0900 h and 1000 h on the first day of the experiment, the animals were moved to individual cages, which were placed below an activity sensor attached to the base of the cage above (20 cm above the floor of the lower cage). Their position was configured to ensure that they monitored the whole area of the cage floor that was accessible to the mice. Animals were allowed to habituate to their individual housing conditions throughout day 1. As a precaution, data collected over this 24 h period were excluded from subsequent analyses. In order to correct for any effect of the position of the cage in the rack, mice from all four test groups were monitored simultaneously and were balanced across five activity sensors.

The sensors are activated by turns of the body and rearing, as well as gross ambulatory movement, but not vegetative movements or those associated with respiration and muscle twitches during sleep. A full description of the design and specifications of the apparatus and software is to be published elsewhere (Brown, *personal communication*). Briefly, these activity-monitoring devices incorporated passive infra-red (PIR) sensors (Panasonic AMN 32111; Premier Farnell UK Limited), which detect the movement of sources of heat. These were used alongside a light-dependent resistor (LDR). The PIR sensors were configured as digital inputs to an Arduino Uno (Rev3) microcontroller board, (http://arduino.cc/en/Main/ArduinoBoardUno) and the state of each PIR was recorded at 100 millisecond intervals. At the end of each 60 second interval, the percentage activity for each PIR sensor was calculated and sent as a serial message alongside a measurement of relative environmental light from the LDR. Serial messages were captured by a laptop, running companion software that saved the data from the sensor, alongside a timestamp, as a tab-delimited text file. Data were then transferred to a Microsoft Excel file for further statistical analysis using InVivoStat (version 2.2.0.0.; Clark *et al.* 2011).

### 5-Choice Serial Reaction-Time Task

Owing to the large number of mice needed for this study (48 in total), the experiment was carried out in two replicate steps, each of which involved the training and testing of 6 mice from each experimental group (*WT-Hom*, *KO-Hom*, *WT-Het*, *KO-Het*). As a precaution, the *WT-Het* and *KO-Het* mice in the second half of the experiment were re-derived from the two inbred strains, rather than using a new batch of descendents of the same heterozygous breeding-pairs. Mice from the *Hom* colony were taken from four breeding-pairs for each genotype (two for each half of the experiment), while mice from the *Het* colony were taken from six breeding-pairs (three for each half of the experiment).

The mice were aged 6–8 weeks and weighed 27–41 g at the start of training (mean age/start weight for each group: *WT-Hom*, 6.5 ± 0.1 weeks/34.55 ± 0.71 g; *KO-Hom*, 6.6 ± 0.1 weeks/31.85 ± 0.53 g; *WT-Het*, 7.9 ± 0.2 weeks/38.40 ± 1.00 g; *KO-Het*, 7.8 ± 0.2 weeks/34.80 ± 0.60 g) and were housed in fixed groups of 2 - 4 littermates per cage, throughout. All animals were subject to restricted food supply to stabilize their body weight at 90% free-feeding weight. They were brought into the laboratory at the same time every day (Monday to Friday: 0900 h to 0930 h) and weighed before training/testing in the 5-CSRTT. Half the animals were trained and tested in the morning (1000 h to 1200 h) while the remainder were trained and tested in the afternoon (1300 h to 1500 h). Animals from the two colonies and two genotypes were balanced across the morning and afternoon sessions.

Details of the protocol are reported fully elsewhere (Dudley *et al.* 2013; Yan *et al.* 2011). In brief, after habituation to the 5-CSRTT apparatus (Med Associates, St. Albans, VT, USA), the animals were trained to nose-poke in response to a light stimulus in one of five holes in one wall of the chamber: a correct response was rewarded with sweetened milk (0.01 ml of 30% condensed milk solution), delivered from a magazine in the opposite wall of the chamber. Animals were trained in a series of six stages and were required to attain specific performance criteria at each stage. These criteria were based on: the total number of trials completed; the number of correct trials completed; number of premature responses; accuracy; and omissions. Successive stages of training were made progressively more difficult by: decreasing the duration of the light stimulus (stimulus duration, ‘SD’); increasing the amount of time the animal had to wait before the light stimulus appears (intertrial interval, ‘ITI’); and decreasing the length of time during which the animal was allowed to respond to the light stimulus (limited hold, ‘LH’). See Table1 for details of the performance variables, task parameters and progression criteria used at each stage of training.

**Table 1:** (a) Task parameters and progression criteria for each stage of the training procedure of the 5-CSRTT. (b) Performance variables measured in the 5-CSRTT

(a)				
	Stage parameters			
Stage	SD (seconds)	LH (seconds)	ITI (seconds)	Progression criteria
1	30	30	2	≥30 correct trials
2	20	20	2	≥30 correct trials
3	10	10	5	≥50 correct trials
4	5	5	5	≥50 correct trials; ≥ 75% accuracy;
				≤25% omissions; total trials – premature = 100
5	2.5	5	5	≥50 correct trials; ≥ 75% accuracy;
				≤25% omissions; total trials – premature = 100
6	1.8	5	5	≥50 correct trials; ≥ 75% accuracy;
				≤25% omissions; total trials – premature = 100

(b)	
Premature responses/100 trials	[premature responses/(correct + incorrect responses + omissions)] × 100
%Omissions	[total omissions/(correct + incorrect responses + omissions)] × 100
Perseveration score	total nose-pokes into the same hole following a correct response
Total trials	correct responses + incorrect responses + omissions
%Accuracy	[correct responses/(correct + incorrect responses)] × 100
Latency to correct response (seconds)	latency to nose-poke into the correct hole after the onset of the light stimulus
Latency to collect reward (seconds)	latency to collect the milk reward following a correct response

ITI, intertrial interval; LH, limited hold; SD, stimulus duration.

Once stable performance had been achieved at Stage 6 of training (‘baseline’ performance: >50 correct trials, >75% accuracy, <25% omissions and total trials = 100), subjects were tested in two ways, so as to challenge different aspects of cognitive performance. One used a fixed, long intertrial interval (LITI), in which the ITI was increased from 5 seconds to 7 seconds. The other used a variable intertrial interval (VITI: 2, 5, 10, 15 seconds), in which the ITIs were delivered in a random sequence. To eliminate any potential carry-over effects of previous experience of the tests, the sequence of testing in the VITI and LITI was counterbalanced across all experimental factors (Colony, Genotype and Time-of-Day). The VITI and LITI tests were carried out on Fridays, only. On the intervening days (Monday–Thursday), the animals carried out Stage 6 of the training procedure, to ensure that baseline performance was restored before the next test day.

By the end of the study, four mice (1 *WT-Hom*, 2 *WT-Het* and 1 *KO-Het*) had not reached the criteria for baseline performance for Stage 6 and so were excluded from all statistical analyses (including training).

### Statistical analysis

Statistical analyses were carried out using InVivoStat (Clark *et al.* 2011). For both experiments, diagnostic plots for normality of the data-set and equality of the variance of the samples were checked and, when necessary, the data were transformed [square-root(score) or Log_10_(score + 1)] to optimize the homogeneity of variance across the four groups. Because the aim of the experiment was to investigate the effects of breeding strategy on pups' behaviour, we defined ‘Colony’ and ‘Genotype’ as fixed factors (i.e. ‘treatments’) in the analysis. We could not assume that all offspring within a single cage would be affected in the same way by these factors (which would include differences such as parental care, interaction with littermates, and/or maternal physiology) and so individual mice, which received these ‘treatments’, were regarded as the experimental unit.

Although Colony was treated as a between-subjects factor, if there was no interaction between Colony and the variable of interest, data for the two colonies were collapsed for analysis of the effect of Genotype, which was treated as another between-subjects factor. Mead's Resource Equation was used routinely to confirm that sample sizes were large enough to detect statistically significant differences.

#### Locomotor activity

Only data collected between days 2 and 7 were included in the statistical analysis (see above). For each animal, data captured at 60-second intervals were grouped into 6 h time bins, in order to compare animals' activity during the first and second half of the light and dark phases (Early Light Phase: 0700 h to 1259 h; Late Light Phase: 1300 h to 1859 h; Early Dark Phase: 1900 h to 0059 h; Late Dark Phase: 0100 h to 0659 h). In order to determine whether activity changed across days 2–7, each 6 h time bin was first analysed using mixed model anova with ‘Day’ as the within-subjects factor and ‘Colony’ and ‘Genotype’ as between-subjects factors. If there was no interaction between Day and Colony and/or Genotype, time-matched data for each 6 h time bin were pooled across days 2–7 to produce a mean activity for that time bin (*N* = 1 for each animal). Mean activity of each colony and genotype was then compared across the different time bins using repeated measures anova with ‘Time Bin’ as the within-subjects factor and ‘Colony’ and ‘Genotype’ as the between-subjects factors. Because changes in locomotor activity during the early dark phase, the late dark phase and early light phase of days 2–7 did not interact with either colony or genotype, the data for these six days were pooled for each time bin. However, during the late light phase, changes in activity over days 2–7 depended on Colony (Colony*Day: *F*_5,80_ = 3.17, *P* = 0.012) and so, for this phase, Day was treated as a separate factor in the analysis.

#### 5-CSRTT

For the training phase, the analysis compared data from the first day of each stage of training, using 4-way mixed model anova, with ‘Colony’, ‘Genotype’ and ‘Time-of-Day’ (morning or afternoon) as the between-subjects factors and ‘Stage (of training)’ as the within-subjects factor. Data from the testing phase of the experiment (VITI and LITI) were first analysed using 3-way single measures anova, with ‘Colony’, ‘Genotype’ and ‘Time-of-Day’ treated as between-subjects factors. *Post hoc* 2-way, 1-way anova or the LSD test were used for more detailed comparisons of specific test groups. Data points that deviated from the mean by more than 3× standard deviations were treated as outliers and removed from analysis. This was necessary only for the VITI, with one mouse eliminated from the analysis of *perseveration* (1× *WT-Hom*), *%accuracy* and *latency to correct response* (1× *KO-Hom*), and *latency to collect reward* (1× *KO-Hom*). As a consequence, the statistical analyses compared groups of *N* = 10-12. Statistical significance was set at *P* < 0.05.

## Results

### Both genetic and environmental/epigenetic factors influence locomotor activity, but at different phases of the 24 h cycle

**Figure 1:**
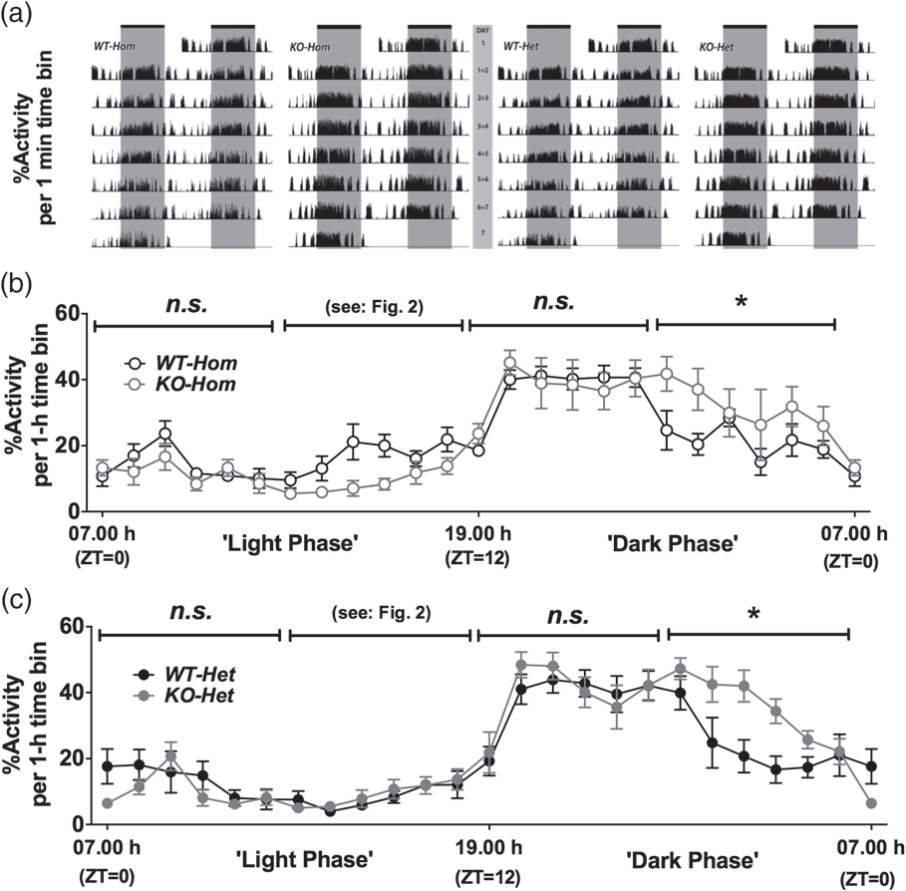
Spontaneous motor activity across the 24 h light:dark cycle. (a) Double-plotted actograms displaying the % activity (per 1 min time bin) of one mouse from each group (*WT-Hom*, *KO-Hom*, *WT-Het*, *KO-Het*) across days 1–7, (b) mean % activity (per 1 h time bin) of *WT-Hom* mice and *KO-Hom* mice across the 24 h cycle, and (c) mean % activity (per 1-h time bin) of *WT-Het* mice and *KO-Het* mice across the 24 h cycle. (a) Areas in white represent the light phase of the 24 h light:dark cycle, whereas areas in grey represent the dark phase of the 24 h light:dark cycle. (b) and (c) Circles depict mean ± SEM for each hourly time-point and lines above the graphs represent 6 h time bins used for statistical analysis. For each individual animal, data from time-matched samples were pooled across six consecutive days to produce the mean ± SEM for each time bin (*N* = 1 for each animal; *N* = 5 per experimental group). The light intensity in the holding room was increased or reduced in steps between 07.00 h and 08.00 h and 19.00 h and 20.00 h, respectively; *P < 0.05; *n.s*., non-significant; ZT, zeitgeber time (light cue).

Double-plotted actograms, showing the activity of one mouse from each group (*WT-Hom*, *KO-Hom*, *WT-Het*, *KO-Het*) across days 1–7, are shown in Fig. 1a.

The activity of the two colonies did not differ during the entire dark phase or the early light phase of the 24 h cycle. However, during the late dark phase, the activity of NK1R-/- mice, from both colonies, was greater than that of their respective wildtypes [Genotype*Time Bin: *F*_2,32_ = 7.97, *P* = 0.002, LSD: (*Hom*): *P* = 0.046 and (*Het*): *P* = 0.031; Fig. 1b,c, respectively].

**Figure 2:**
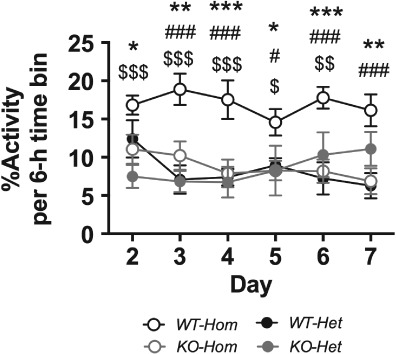
Spontaneous motor activity during the late light phase of the light:dark cycle across days 2−7. Circles depict mean ± SEM for this time bin across days 2–7 (*N* = 1 for each animal; *N* = 5 per experimental group). *P < 0.05, **P < 0.01, ***P < 0.05 (cf. *WT-Hom* vs. *KO-Hom*); #*P* < 0.05, ##*P* < 0.01, ###*P* < 0.001 (cf. *WT-Hom* vs. *WT-Het*); $*P* < 0.05, $$*P* < 0.01, $$$*P* < 0.001 (cf. *WT-Hom* vs. *KO-Het*).

During the late light phase, differences in the activity of the two colonies changed over days 2–7 and interacted with genotype (Fig. 2). This is because the activity of both *KO-Hom* and *WT-Het* mice decreased over this time (LSD: *P* < 0.05 for all), whereas the activity of *KO-Het* mice progressively increased (LSD: *P* < 0.05 for all). Notwithstanding these changes, the activity of *WT-Hom* mice, throughout days 2-7, was greater than that of all other groups of mice (LSD: *P* < 0.05 for all), which did not differ from each other.

### NK1R-/- mice from both colonies display a higher incidence of premature responses and perseveration during training in the 5-CSRTT

**Figure 3:**
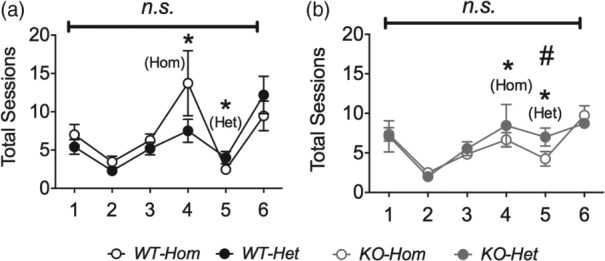
Total sessions required to pass training. Lines above graphs indicate colony comparisons. Circles show mean ± SEM. Numbers below graphs indicate stage of training. *P < 0.05 (cf. WT vs. KO); #*P* < 0.05 (cf. *Hom* vs. *Het*); *n.s*., non-significant. *N* = 10–12 per group.

**Figure 4:**
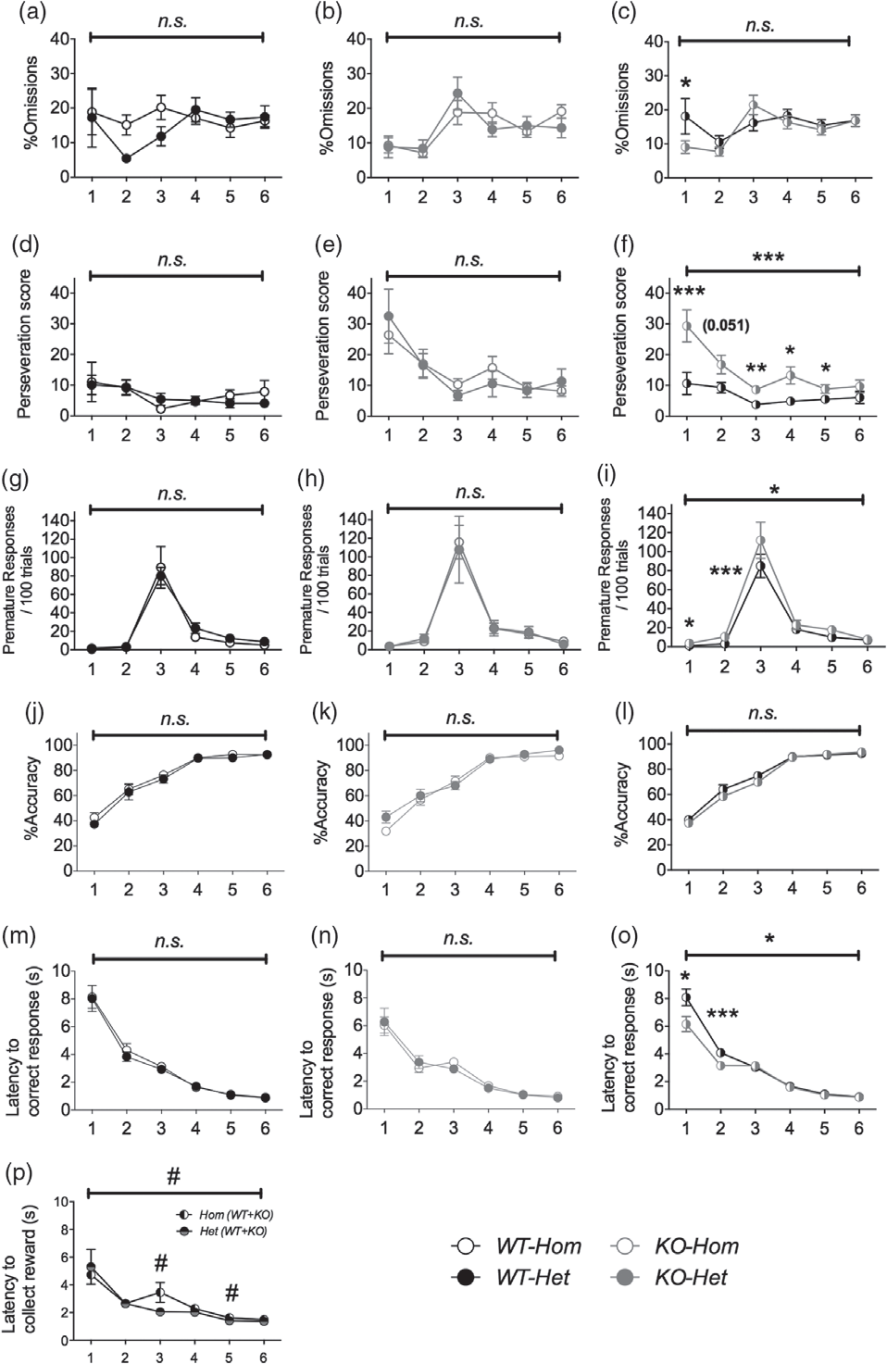
Training in the 5-Choice Serial Reaction-Time Task. There was no main effect of Colony on *%omissions* (a and b), *perseveration* (d and e) or *premature responses* (g and h). The effect of Genotype on *%omissions* depended on Stage: wildtypes showed greater *%omissions* than NK1R-/- mice at stage 1 but not at any later stages of training (c). Overall, *perseveration* (f) *and premature responses* (i) were greater in NK1R-/- mice compared with wildtype mice. *%Accuracy* did not differ in the two colonies (j and k) or the two genotypes (l). *Latency to correct response* also did not differ in the two colonies (m and n), but was greater in wildtype mice compared with NK1R-/- mice overall, although this depended on stage (o). *Latency to collect reward* differed in the two colonies, with mice from the *Hom* colony taking longer than mice from the *Het* colony (p). Lines above graphs indicate comparisons between the two colonies or genotypes. Circles show mean ± SEM. Numbers below graphs indicate stage of training. *P < 0.05, **P < 0.01, ***P < 0.001; *n.s*., non-significant; *N* = 10–12 per group.

During the training phase, there was no overall difference in the *total number of sessions* needed for the two colonies to reach the graduation criteria (Fig. 3a,b). Of all the behaviours measured, only *latency to collect the reward*, which was slightly longer in *Hom* mice (*F*_1,36_ = 4.34, *P* = 0.044, Fig. 4p), differed in the two colonies (Fig. 4).

When comparing genotypes on the first day of each Stage of training, NK1R-/- mice from both colonies showed a higher incidence of *perseveration* (*F*_1,36_ = 15.53, *P* < 0.001, Fig. 4f) and *premature responses* (*F*_1,36_ = 6.00, *P* = 0.019, Fig. 4i). There was no overall genotype difference in *%omissions*, despite a greater incidence of this deficit in wildtypes from both colonies during Stage 1 (*F*_5,180_ = 2.33, *P* = 0.044, Fig. 4c). *Latency to correct response* was also longer in wildtypes from both colonies (*F*_1,36_ = 5.63, *P* = 0.023), especially during the early stages of training (*F*_5,180_ = 3.31, P < 0.007, Fig. 4o).

At the end of Stage 6 of training, there were no colony or genotype differences for any of the behavioural measures.

### The incidence of premature responding, but not omissions or perseveration, during the VITI test differs in NK1R-/- mice from the Hom and Het colonies

**Figure 5:**
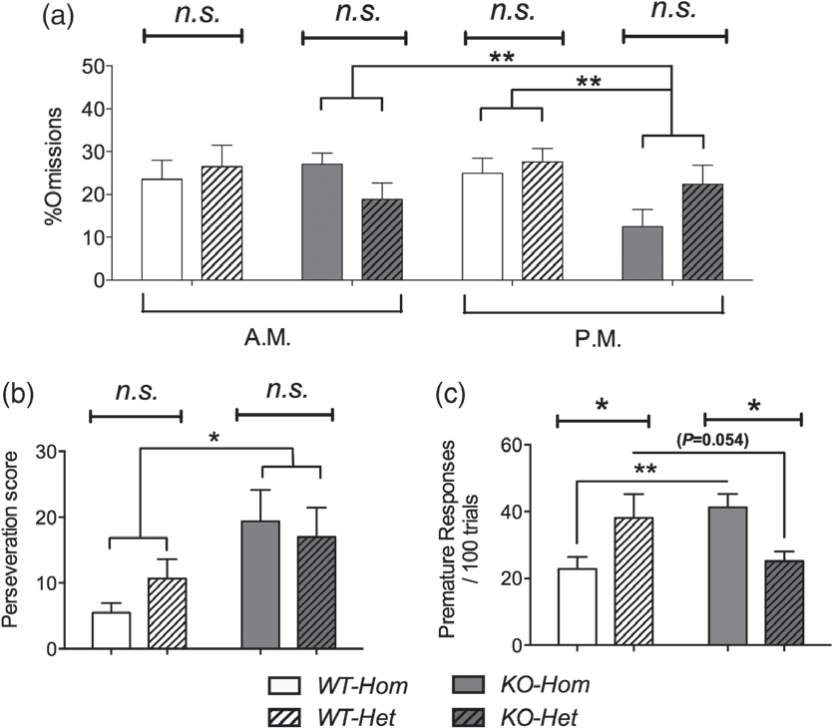
Variable intertrial interval (VITI) in the 5-Choice Serial Reaction-Time Task: attention, perseveration and impulsivity. There was no main effect of colony on either *%omissions* or *perseveration* (a and b). However, *%omissions* were lower in NK1R-/- mice when tested in the afternoon (a) and NK1R-/- mice displayed greater *perseveration* than wildtype mice, overall (b). The effect of genotype on *premature responses* depended on colony: in the *Homs* colony, *premature responses* were greater in NK1R-/- mice compared with wildtypes, whereas this was not the case for the *Het* colony (c). Lines indicate comparisons between the main factors or groups. Bars show mean ± SEM. *P < 0.05, **P < 0.01; *n.s*., non-significant. *N* = 10–12 per group.

Neither *%omissions* nor *perseveration* differed in the two colonies in the VITI test (Fig. 5a,b). However, *perseveration* was greater in NK1R-/- mice, from both colonies, compared with their wildtypes (*F*_1,40_ = 5.47, *P* = 0.024). There were also genotype differences in *%omissions*, but these depended on whether animals were tested in the morning or afternoon (Genotype*Time of day: *F*_1,39_ = 8.28, *P* = 0.006). Specifically, the two genotypes did not differ when tested in the morning but, in the afternoon, *%omissions* were higher in wildtypes than NK1R-/- mice (LSD: *P* = 0.001).

Genotype differences in *premature responses* depended on Colony (Genotype*Colony: *F*_1,39_ = 11.98, *P* = 0.001, Fig. 5c). As in previous studies, *KO-Hom* mice expressed more *premature responses* than *WT-Hom* mice (LSD: *P* = 0.006), but *KO-Het* mice just missed the criterion for carrying out fewer *premature responses* than *WT-Het* mice (LSD: *P* = 0.054).

**Figure 6:**
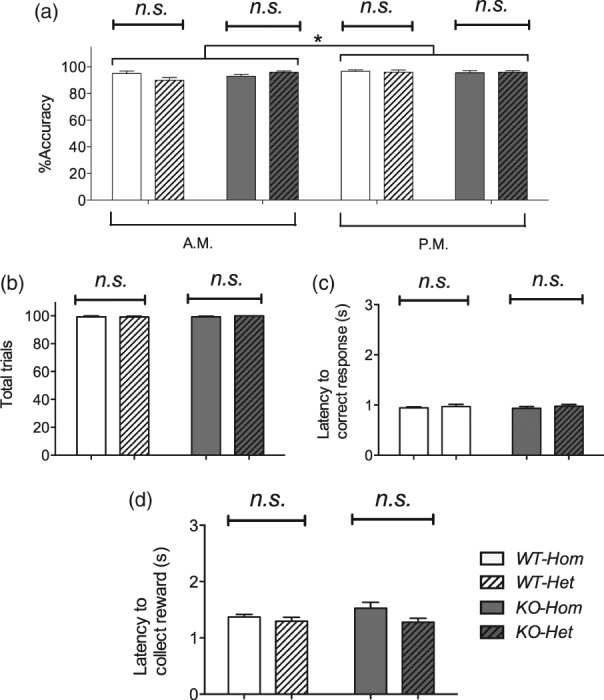
VITI in the 5-Choice Serial Reaction-Time Task: accuracy, total trials and latencies. *%Accuracy* was slightly higher when animals were tested in the afternoon compared with the morning (a). There was no main effect of any of the main factors on *total trials* (b), *latency to correct response* (c) or *latency to collect reward* (d). Lines indicate comparisons between the main factors or groups. Bars show mean ± SEM. *P < 0.05, **P < 0.01, ***P < 0.001; *n.s*., non-significant. *N* = 10–12 per group.

There were no colony differences in *%accuracy*, *total number of trials completed*, *latency to correct response* or *latency to collect reward* in the VITI test (Fig. 6a–d) and none of these measures differed in the two genotypes. Overall, *%accuracy* was slightly higher (c. 3%) in the afternoon than in the morning (*F*_1,34_ = 5.05, *P* = 0.031, Fig. 6a).

### The behaviour of NK1R-/- mice from Hom and Het colonies does not differ in the LITI test

**Figure 7:**
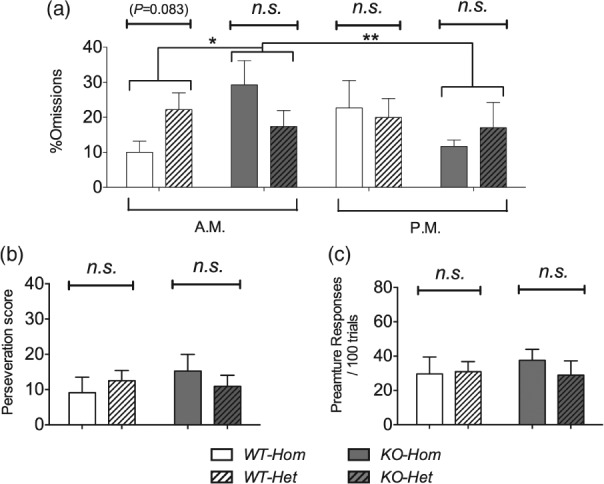
Long intertrial interval in the 5-Choice Serial Reaction-Time Task: attention, perseveration, impulsivity. *%Omissions* did not differ in the two colonies (a). *%Omissions* were greater in NK1R-/- mice when tested in the morning but not in the afternoon. There was no main effect of colony, genotype or time-of-day on *perseveration* (b) or *premature responses* (c). Bars show mean ± SEM. Lines indicate comparisons between the main factors or groups. *P < 0.05, **P < 0.01, ***P < 0.001; *n.s*., non-significant*. N* = 10–12 per group.

When the two colonies were tested with the LITI, there was no difference in *%omissions*, *perseveration* or *premature responses* (Fig. 7a–c). *Perseveration* and *premature responses* also did not differ in the two genotypes. However, as in the VITI (see above), a genotype difference in *%omissions* depended on time of day (Genotype*Time of day: *F*_1,39_ = 8.51, *P* = 0.006). Regardless of colony, *%omissions* were greater in NK1R-/- mice than their wildtypes when they were tested in the morning (LSD: *P* = 0.035). Moreover, *%omissions* by NK1R-/- mice was greater in the morning than the afternoon (LSD: *P* = 0.003).

**Figure 8:**
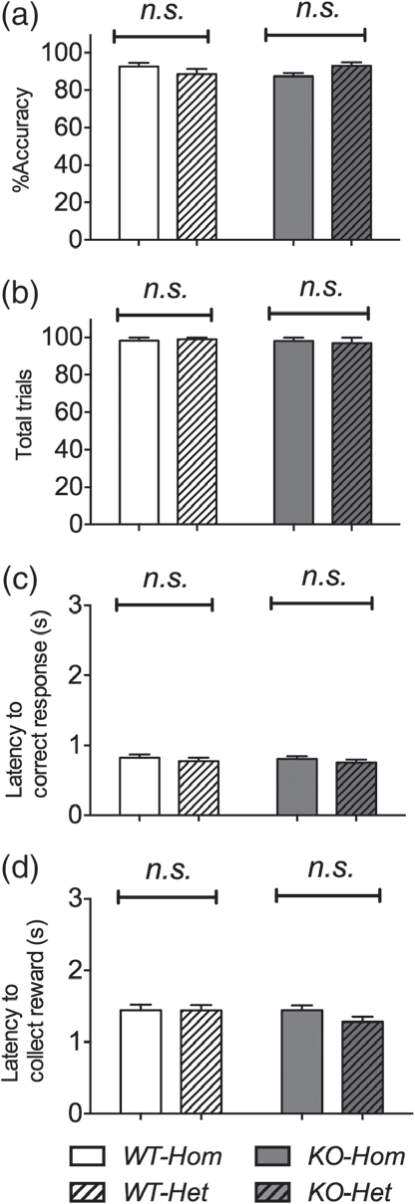
Long intertrial interval in the 5-Choice Serial Reaction-Time Task: accuracy, total trials and latencies. None of these behavioural measures differed in the two colonies. There was also no main effect of any of the other experimental factors (a–d). Lines at top of graphs indicate colony comparisons. Bars show mean ± SEM. *P < 0.05, **P < 0.01, ***P < 0.001; *n.s*., non-significant. *N* = 10–12 per group.

As in the VITI test, there were no colony differences in *%accuracy*, *total number of trials completed, latency to correct response,* or *latency to collect reward* and none of these measures differed in the two genotypes (Fig. 8a–d).

## Discussion

### Genotype and breeding environment influence different aspects of the 24 h cycle of motor activity

Both colonies of NK1R-/- mice were more active than their respective wildtypes during the late dark (active) phase of the 24 h cycle. This finding is in line with our report that acute treatment with an NK1R antagonist induced hyperactivity in inbred wildtypes (Yan *et al.* 2010) and suggests that a lack of functional NK1R, alone, is sufficient to induce hyperactivity.

A second finding was that the diurnal regulation of motor activity was disrupted in NK1R-/- mice. There are many reasons to expect abnormal light-entrained activity rhythms when NK1R function is impaired. Light activates the retinohypothalamic tract (RHT), which projects to the suprachiasmatic nucleus (SCN), directly, and indirectly via the thalamic intergeniculate leaflet (‘IGL’; Morin *et al.* 2003). Substance P is found within the core and/or in peripheral zones of the SCN (Hartwich *et al.* 1994; Piggins *et al.* 2001) and both substance P and NK1R are expressed in the IGL (Morin *et al.* 1992; Piggins *et al.* 2001). NK1R antagonism suppresses excitatory postsynaptic currents in the SCN, evoked by optic nerve stimulation (Kim *et al.* 1999, 2001), whereas treatment with an NK1R antagonist selectively blocks the ability of light to induce phase advances of locomotor activity (Challet *et al.* 2001). It is interesting that phase advances occur in response to light exposure during the late active phase, which is when we observed differences in motor activity in NK1R-/- mice.

NK1R are also expressed by some serotonergic neurones in the raphé nuclei (Lacoste *et al.* 2006), which is thought to gate motor activity (Jacobs & Azmitia 1992). Central serotonergic transmission contributes to motor entrainment of the circadian rhythm (Edgar *et al.* 1997; Meyer-Bernstein *et al.* 1997), most likely through activation of serotonergic receptors in the SCN (e.g. Horikawa *et al.* 2000). It follows that the abnormal serotonergic transmission in NK1R-/- mice (Froger *et al.* 2001) could also help to explain this disruption of their 24 h motor rhythm.

A third finding was that the activity of *WT-Hom* mice during the late light phase was greater than that of all other groups of mice. This difference depends on functional NK1R because these *WT-Hom* mice were also more active than the *KO-Homs* at this time. Yet, there was no difference in the activity of *WT-Het* and *KO-Het* mice and so we infer that environmental and/or epigenetic factors either blunted the activity of the *WT-Hets* or increased that of *WT-Homs*. There is extensive evidence that early-life experience affects adult behaviour (see Meaney 2010). Epidemiological studies also suggest that a range of environmental factors interact with a genetic vulnerability for ADHD (see Elia *et al.* 2014).

The abnormal diurnal motor rhythm of NK1R-/- mice has a clear equivalent in ADHD. Many patients experience delayed sleep onset at the end of the active phase (Kooij & Bijlenga 2013; van Veen *et al.* 2010) while some ADHD patients also experience delayed waking at the end of the resting phase (Cortese *et al.* 2013). Here, an obvious difference is that mice from the *Hom* colony were bred, weaned and housed separately, but mice from the *Het* colony were bred and weaned by NK1R+/− dams and housed in mixed litters. The extent to which interactions between pups and their mother and/or littermates account for the differences in behavioural phenotypes of the two colonies merits further investigation. Such studies could shed light on whether an interaction between early-life experiences and polymorphism(s) of *TACR1* disrupt motor rhythms in ADHD patients.

### Impulsivity of NK1R-/- mice in the 5-CSRTT (VITI) is influenced by environmental/epigenetic factors

NK1R-/- mice, from either colony, carried out more *premature responses* overall than their wildtypes, especially during the early stages of training. This finding points to a lack of functional NK1R as a factor that can exacerbate impulsivity. Because there were no differences in any behavioural measure at the end of training, it seems that neither the two colonies nor two genotypes differed in their ability, or their motivation, to carry out the task at this stage of the protocol. Nonetheless, we cannot be certain that the food restriction increased motivation to the same extent in the two colonies/genotypes.

When tested in the VITI, *KO-Hom* mice carried out more *premature responses* compared with their wildtypes. Because this increase did not occur in the LITI, the greater incidence of *premature responses* in *KO-Hom* mice seems to depend on the stimulus being unpredictable, rather than an increase in motivation to respond. Whatever the explanation, it is striking that there was no increase in *premature responses* in *KO-Het* mice. These findings suggest that the incidence of this behaviour is determined by an interaction between NK1R and other (e.g. environmental and/or epigenetic) factors.

There is extensive evidence for interactions between mother and pups that influence cognitive performance (e.g. Hao *et al.* 2011). Early-life experiences also affect susceptibility to psychiatric disorders in later life (Anda *et al.* 2006; Lima *et al.* 2010). As yet, there is no evidence that epigenetic changes affect NK1R (*TACR1*) function. However, repeated cocaine administration does decrease DNA methylation of the *TACR3* gene (*Nk3r* gene in mice: Barros *et al.* 2013). Detailed comparison of the influence of breeding strategy on the behaviour of NK1R-/- mice in the two colonies could help to identify the cause(s) of excessive impulsivity.

### Perseveration and impaired attentional performance of NK1R-/- mice are a direct consequence of a lack of NK1R

*Perseveration* did not differ in the two colonies but was higher in NK1R-/- mice during training and the VITI test, as in our previous studies (Dudley *et al.* 2013; Yan *et al.* 2011). These findings suggest that this behaviour is attributed directly to a lack of functional NK1R. Importantly, they rule out environmental/epigenetic influences on *perseveration*, in the VITI at least. It is striking that repetitive behaviour (including compulsive checking) is evident in some ADHD patients (Gürkan *et al.* 2010). The lack of any genotype difference in the LITI test fits with our experience that the incidence of *perseveration* in the LITI is more variable than in the VITI (cf. Dudley *et al.* 2013; Yan *et al.* 2011), suggesting that the LITI and VITI tests must recruit different neuronal processes.

There was no difference in *%omissions* in the two colonies during training or testing and so we infer that epigenetic factors have no bearing on this behaviour. Interestingly, genotype-dependent differences in this behaviour depended on time of day, as in our previous study (Yan *et al.* 2011). There is evidence for a circadian rhythm in cognitive performance (e.g. Winocur & Hasher 2004). Performance deficits in ADHD patients similarly depend on time of day (Usami *et al*. 2013). However, we cannot distinguish whether the changes we report are explained by a disruption of a circadian influence or an extraneous procedural factor. They are unlikely to arise from any genotype-dependent change in motivation to respond because neither the *latency to correct response* nor *latency to collect the reward* differed in the morning and afternoon. Whatever the explanation, we infer that this behaviour is not influenced by the breeding environment.

We did not explore the mechanisms that could underlie these behavioural abnormalities. However, a lack of NK1R would blunt both glutamatergic excitation and GABAergic inhibition of noradrenergic projections from the locus coeruleus to the prefrontal cortex, which govern visual attention (see Yan *et al*. 2009). NK1R are also densely expressed on (cholinergic) interneurons in the striatum (Gerfen 1991), where they mediate the release of acetylcholine and dopamine (Galarraga *et al.* 1999), both of which have been implicated in impulsive behaviour (see Jupp & Dalley 2014; Moreno *et al.* 2013). The functional integrity of these interneurons is thought to determine appropriate motor responses to salient environmental stimuli (e.g. Ding *et al.* 2010) and to constrain *impulsivity* and *perseveration* (e.g. Burguière *et al*. 2013; Christakou *et al.* 2004; Chudasama *et al.* 2003).

## Conclusions

We infer that the *hyperactivity* and *perseveration* of NK1R-/- mice are a direct consequence of a lack of functional NK1R, which also disrupts the diurnal regulation of motor activity. Greater *%omissions* were evident in NK1R-/- mice only in the morning of the LITI test, indicating that evaluation of this behavioural deficit is confounded by procedural or temporal factors, as yet unidentified. By contrast, the greater *impulsivity* of NK1R-/- mice was influenced by an interaction between genetic and environmental and/or epigenetic factors in combination with a lack of functional NK1R, the effects of which depend on previous test experience and cognitive context.

This study also reveals that differences in the phenotype of genetically-altered animals can be assigned to genetic, environmental/epigenetic factors, or both, only after head-to-head comparisons of the progeny of homozygous and heterozygous breeding-pairs. It follows that biomarkers for ADHD could differ for each behavioural aspect of the disorder (‘endophenotype’). This is particularly important for research into the aetiology of ADHD because there is strong evidence that both genetic (Faraone *et al.* 2005) and environmental factors (including maternal behaviour; Elia *et al.* 2014) increase vulnerability to this disorder.
